# The psychological impact of adult-onset craniopharyngioma: A follow-up survey

**DOI:** 10.1007/s12020-025-04497-0

**Published:** 2026-02-16

**Authors:** Katie Daughters

**Affiliations:** Department of Psychology, University of Essex, Colchester, UK

**Keywords:** Adult-onset craniopharyngioma, Mental health, Psychological wellbeing, Physical wellbeing, Social behaviour

## Abstract

**Purpose:**

Adult-onset craniopharyngioma (AoC) is a rare benign tumour of the sellar/parasellar region associated with significant physical morbidity and a poorer quality of life. Limited medical studies have documented the *psychological* impact of AoC, but psychological studies may be better placed to investigate these effects. This study used an exploratory sequential mixed-methods design to develop and administer a new patient-informed survey to quantitatively assess the psychosocial consequences of AoC.

**Methods:**

A 30-item questionnaire was developed based on previous qualitative interviews with AoC patients and clinicians. Items covered psychological and physical impacts, including mood, social functioning, and emotion regulation. Participants (*N* = 25) were recruited online via a UK-based charity and completed the survey through Qualtrics. Percentage endorsements were calculated for each item, and regression analyses examined associations with individual history.

**Results:**

There was a clear psychosocial impact of AoC: 88% reported low mood, 68% felt they no longer recognized themselves, and 56% missed social events due to anxiety. Physical symptoms were also prominent, but importantly these were associated with significant psychosocial implications. Finally, growth hormone replacement therapy was significantly associated with reduced physical impact.

**Conclusion:**

Findings demonstrate a significant psychosocial impact of AoC, highlighting the importance of integrated mental health care in its management and the need for further psychosocial research to improve quality of life for patients.

**Supplementary Information:**

The online version contains supplementary material available at 10.1007/s12020-025-04497-0.

## Introduction

Adult-onset craniopharyngioma (AoC) is a rare benign brain tumour that grows in the sellar and parasellar region, severely disrupting critical structures in this area including the optic nerve, pituitary gland and hypothalamus [1]. Advances in treatment and guidelines has greatly improved the mortality rate in recent decades [1–3], however, patients often face life-long physical complications [1] and report a poorer quality of life [4]. Crucially, recent medical research has demonstrated that some of this is related to psychological factors [5–8]. To date, however, there has been only a small handful of *psychological* studies investigating this impact.

Three studies have investigated the psychological consequences of AoC on theory-of-mind and emotion recognition using experimental tasks [9–11]. All studies found that AoC (or AoC and a combination of pituitary tumour) patients performed significantly worse on emotion recognition tasks compared to healthy volunteers, but there were mixed results on the theory-of-mind task. A final study used well-validated psychological questionnaires to assess depression, anxiety and emotional skills and found AoC patients reported significantly higher levels of depression and anxiety compared to healthy volunteers, but no difference in emotional skills [12].

In combination with the small body of medical studies, the above studies suggest that patients with AoC experience various psychological impacts from their diagnosis or treatment. While psychological questionnaires and experimental tasks provide significant quantitative insight into these particular phenomena, like all quantitative approaches they are limited in capturing diverse yet detailed information. In contrast, qualitative research can provide a rich, well rounded, and in-depth understanding of the lived experience of patients with medical conditions [13]. Our recent qualitative study [14] sought to provide this rich insight into AoC patients’ experience and identified two key themes; (1) that patients do experience a psychological impact of AoC, and (2) that they experience common physical symptoms.

Within the first theme, and consistent with previous literature [5, 7, 12], participants reported core psychological symptoms, predominantly depression and anxiety. Importantly, participants also provided a more nuanced understanding of how AoC, and the subsequent treatment, had a much broader and subtler effect on their psychological functioning. Social impairments included difficultly with social interactions, a shrinking social network and poorer emotion regulation which negatively impacted relationships. Protective factors were also identified, including maintaining a level of independence, the ability to work and leveraging mental resilience [14].

The second theme centred on physical symptoms, and their strong psychological impacts [14]. Consistent with medical literature [1, 4], patients reported a small set of core symptoms (weight-gain and fatigue) and a set of secondary symptoms (low libido, difficulty managing diabetes insipidus related symptoms, and more frequent headaches) that varied more across patients. Crucially, the psychological consequences of these symptoms were important, including lower self-esteem, increased self-consciousness, and fewer or more negative social interactions with friends and family.

This qualitative study [14] provided much-needed detail to the existing literature, identifying key areas of social and emotional strengths and difficulties for AoC patients. The current study sought to follow-up this original research in an exploratory sequential mixed-methods design whereby qualitative research is first used to consult the patient group and define the research area, and quantitative research is used to determine the generalisability of the findings in a larger, community-based sample [15, 16]. Thus, the current study created a new questionnaire based on the findings of the qualitative study [14] and sought to recruit as many participants as possible via an online survey. The aim of the study was to quantitatively assess the endorsement of statements identified in the qualitative research.

## Materials and methods

### Participants

Participants were recruited via an advert hosted by the national charity, The Pituitary Foundation, across their social media platforms. The Pituitary Foundation is the leading source of information and support for patients and families with pituitary conditions in the UK and regularly advertise research studies to their followers (14000 followers on Facebook; 7500 followers on X (as of September 2025)).

**Fig. 1:**
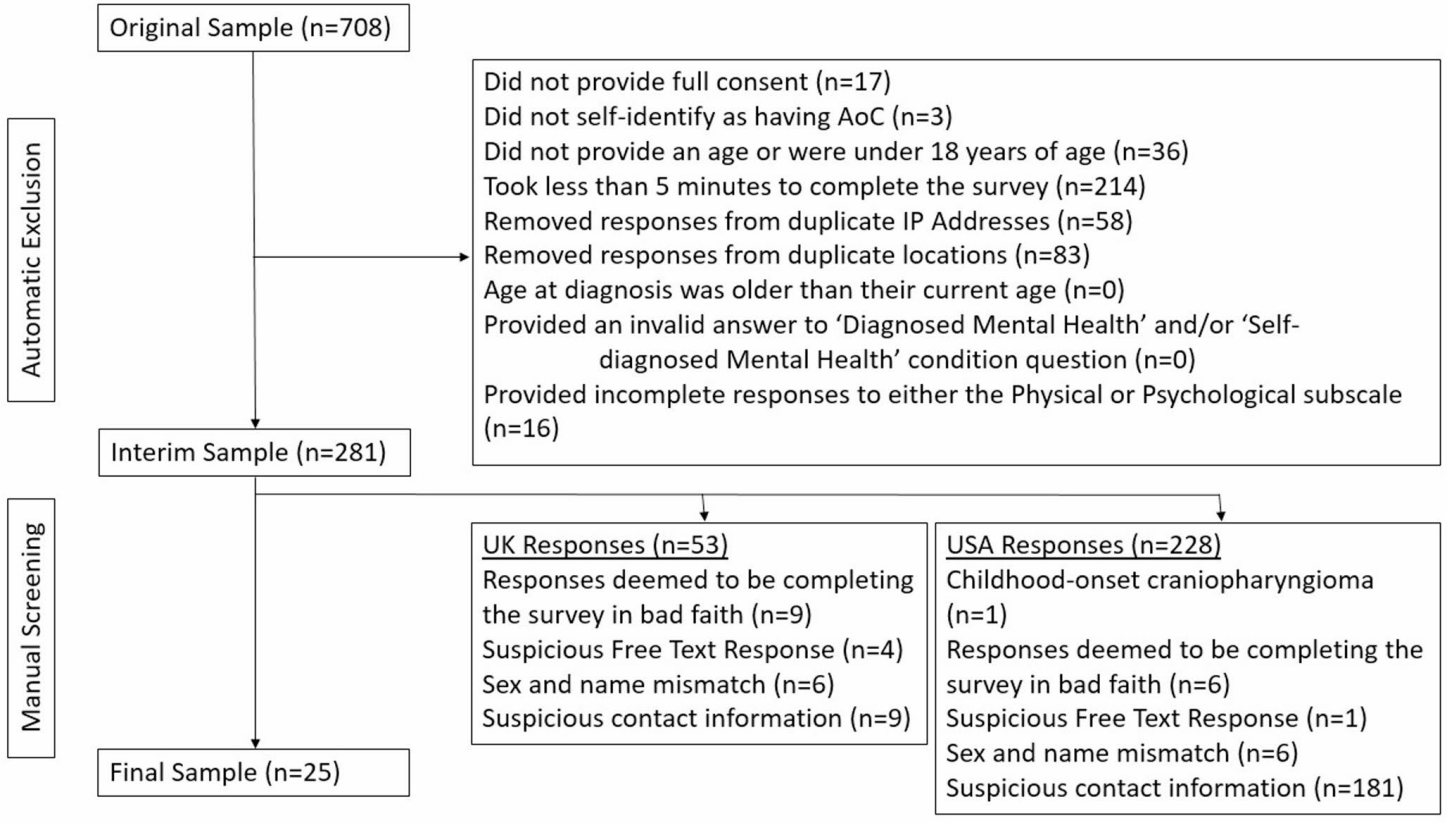
CONSORT diagram illustrating the process and order of screening criteria applied to the original dataset and the number of participants excluded at each step

The advert posted on X received over 4500 views. The study link was accessed 708 times on the day it was posted, and the study was closed for recruitment. In accordance with standard screening procedures and considering the significant response a careful screening was undertaken. A flow of participants through this process and drop out numbers are provided in the CONSORT diagram (Fig. 1). The first stage of screening was done automatically. First, participants who did not progress to the survey were removed: non-consent; under 18 or not confirming age; and did not self-identify as having AoC. This left 652 eligible participants. To ensure the quality of the data several steps were taken to screen for careless responding, based on standard practice for online studies [17–19]. Responses were excluded if they met any of the following criteria: survey completion < 5 min; duplicate IP Address or exact same location; diagnosis age older than current age; nonsensical free text responses; and < 100% completion of survey items. This left 281 remaining participants.

The third phase of screening employed manual screening to assess the free text responses and consistency across information provided. On reviewing the data, it was noticed that responses either came from the UK or USA. Given that the study was designed to follow-up in-depth qualitative results from UK patients the dataset was split by location. After final screening, the UK dataset had 25 participants, and the USA dataset had 33 participants (see Fig. 1). Unfortunately, several observations of the USA dataset did not meet satisfactory standards, and the decision was taken to remove these data from the analyses. For transparency, the USA dataset and code are available online (see below) and a brief discussion of the results, including reasons for exclusion, are included in Supplemental Information 1. Thus, the current study will present the UK dataset, as a follow-up to the UK-based qualitative results. A summary of the final sample characteristics is provided in Table 1. The study was approved by the Ethics Committee at the University of Essex (ETH2122-0216); all the data and code are available online (https://osf.io/3wrpy/?view_only=9a08260f4f9849b48fc721acc788aaff).

**Table 1:** Clinical characteristics of the final sample

Characteristic	
Age (years)	49 (36–59)
Age at Diagnosis (years)	44 (22–55)
Sex	12 (48%) Male 13 (52%) Female
Undergone Surgical Resection	21 (84%)
Undergone Radiotherapy	20 (80%)
Taking Hormone Replacement	21 (84%)
Diagnosed Diabetes Insipidus	19 (90%)
Taking Growth Hormone	20 (95%)
Diagnosed Mental Health Condition	11 (44%)
Self-Diagnosed Mental Health Condition	9 (36%)

### Materials

The newly created questionnaire was developed based on findings from semi-structured interviews with AoC patients and clinicians who work closely with AoC patients [14]. Items were created that represented important codes from each of the two themes identified from interviews. A code was deemed to be important if it was endorsed by multiple individuals and emphasised throughout interviews. This resulted in 30 items: 20 items relating to psychological symptoms, eight items relating to physical symptoms of AoC, and two items relating to the importance and usefulness of further research (see Table 2). To limit acquiescence bias [20–22], 3 items from the physical and 10 items from the psychological subscales were reverse worded (e.g., the code ‘My weight gain has affected my self-esteem’ was changed to ‘My weight gain has not affected my self-esteem’). For each item, participants had to select one answer on a 5-point scale from “Definitely Disagree” to “Definitely Agree”. All subscale items were presented together and in the order shown in Table 2. Whether participants responded to the physical and psychological subscale first was randomised across participants, however, all participants answered the two items relating to research last. The survey was presented using the software Qualtrics (https://www.qualtrics.com).

### Procedure

Participants were required to read the study information sheet and provided informed consent, as well as confirm that they self-identified as having a diagnosis of AoC. If a participant declined a consent item or did not identify as having AoC they were directed to the end of the survey and could not take part.

Participants provided demographic information (age and sex) as well as a basic medical history including: age at diagnosis, whether they had undergone surgery, had radiotherapy, currently taking hormone replacements, currently taking medication for diabetes insipidus, currently taking growth hormone, had been officially diagnosed with a mental health condition, or self-diagnosed with a mental health condition. Participants then completed the survey, before being invited to claim a £10 voucher and thanked for their participation.

### Data analysis

Cronbach’s Alpha was calculated for each subscale to confirm that the items created a coherent concept. Percentage endorsement for each item was calculated. Multiple linear regressions were carried out to investigate the impact of individual history (predictors were dummy coded) on endorsement of the psychological and physical subscales.

## Results

### Reliability

Both the psychological (α = 0.71) and physical (α = 0.62) subscale achieved good internal consistency scores, indicating that the items of each subscale are a good reflection of the underlying concepts developed from the qualitative interviews. There was no improvement in reliability if any items were dropped, indicating that all items performed well.

### Percentage endorsement

The percentage item endorsement was calculated by summing endorsement for either ‘definitely agree’ and ‘agree’ for regular items and ‘disagree’ and ‘definitely disagree’ for reverse worded items. For ease of interpretation, Table 2 presents just these values for each item. A full table for all participant responses is provided in Supplemental Information 2.

There was strong endorsement of a psychological impact of AoC. An overwhelming majority (88%) reported experiencing low mood, and 68% said they do not feel like themselves anymore. More than half (56%) reported missing social events due to anxiety. The majority said they were good at recognising their own (68%) and others’ emotions (64%), yet 36% also reported crying for no reason. There were significant changes in their relationships, with 68% reporting a difference with their partner and 44% reporting a shrinking social network. Notably, almost half (48%) had discussed their psychological wellbeing with their endocrinologist, and a similar number had been referred for further support (44%).

There was also strong endorsement for physical consequences of AoC. The vast majority reported weight gain (84%) and fatigue (52%), with headaches (72%) and libido (48%) also commonly affected. Crucially, there was a significant psychological impact of these symptoms, with 64% having to modify their lifestyle due to their weight gain and 40% missing social events due to fatigue.

Finally, participants strongly supported further research into the psychosocial aspects of AoC, with 72% finding more information helpful.

**Table 2:** Percentage endorsement for each item in the survey

Theme 1 – Psychological Impact of AoC	
**I have experienced periods of low mood.**	88
**I don’t feel like myself anymore.**	68
I have not felt anxious.*	48
I’m confident in social situations.*	28
**I have missed social events due to anxiety.**	56
I have never experienced ‘brain fog’.*	36
I’m good at recognising other people’s emotions.*	20
I’m good at recognising my own emotions.*	20
**I often cry without really knowing why.**	40
**I was more emotional in the period around my surgery**,** compared to now.**	48
My number of friends has not changed.*	44
My interactions with my friends are exactly the same.*	40
**I see my friends less often than I used to.**	48
**My relationship with my partner has changed.**	68
**If I feel down**, I pick myself up and carry on.*	36
I have not felt depressed.*	40
I find it easy to cope with having a long-life condition.*	32
**I have discussed my psychological wellbeing with my endocrinologist.**	48
**My endocrinologist has asked about my psychological wellbeing.**	60
I have not been referred for mental health support.*	44
**Theme 2 – Physical Impact of AoC**	
**I have experienced weight gain.**	84
**I have had to make changes to my normal lifestyle because of my diagnosis/treatment-related weight gain.**	64
My weight gain has not affected my self-esteem.*	36
I have not experienced fatigue.*	52
**I have missed social events because I am too tired to attend.**	40
**I have experienced low libido.**	48
I enjoy being intimate with my partner.*	12
**I experience more headaches than I used to.**	72
**Theme 3 – AoC Research**	
**I would find more information on the psychological impacts of adult-onset craniopharyngioma interesting.**	68
**I would find more information on the psychological impacts of adult-onset craniopharyngioma useful.**	72

*Items that were reverse scored

### Individual history

Although participants’ individual history could explain 58.3% of variance in their total psychological impact score, the overall model was not significant (*R*^2^ = 0.58, *F*(9,11) = 1.71, *p* = .198) and there were no significant predictors (see Table 3). Participants’ individual history could explain 52.6% of the variance in their physical impact score (Table 4); although the overall model was not significant (*R*^2^ = 0.53, *F*(7,13) = 2.06, *p* = .124), growth hormone replacement therapy (GHRT) significantly predicted a lower physical impact score, indicating that GHRT reduced symptoms.

**Table 3:** The influence of participants’ individual history on their reported psychological impact of AoC

Predictor	β	*p*
Age	-1.26	0.155
Age at Diagnosis	1.71	0.111
Sex	− 0.25	0.395
DI	− 0.27	0.398
GH	− 0.67	0.179
Surgery	− 0.05	0.822
Radiotherapy	0.30	0.217
Diagnosed Mental Health Condition	0.17	0.591
Self-diagnosed Mental Health Condition	0.18	0.433

DI = Diabetes Insipidus; GH = Growth Hormone

**Table 4:** The influence of participants’ individual history on their reported physical impact of AoC

Predictor	β	*p*
Age	− 0.69	0.405
Age at Diagnosis	1.13	0.250
Sex	− 0.29	0.187
DI	− 0.27	0.378
GH	-1.09	**0.032**
Surgery	− 0.16	0.442
Radiotherapy	0.12	0.583

*Values bold represent significant results

DI = Diabetes Insipidus; GH = Growth Hormone

## Discussion

The present study aimed to quantitatively assess the psychological impact of AoC using a new survey informed by recent qualitative research, determining the extent to which the lived experiences of those patients are endorsed by a larger, community-based sample.

While the results confirm previous research [1, 3, 5, 7] indicating that AoC patients are at high-risk of developing mood disorders and experience significant weight-gain and fatigue, they also reveal a range of more subtle effects impacting on patients’ social lives and wellbeing. These effects include, a loss of sense-of-self and poorer self-esteem, negative impacts on romantic relationships and friendships, and reduced opportunities for social contact (due to anxiety and fatigue) and a subsequent reduction in social network size.

One intriguing finding is the high endorsement of patients’ ability to recognise their own and others’ emotions. This contrasts with previous studies which found that AoC patients performed significantly worse on emotion recognition tasks compared to healthy volunteers [9–11], but in line with a previous study [12] that found no differences when using a psychological questionnaire. This raises an interesting question about subjective versus objective measures with respect to capturing emotional experience in AoC patients. Future research should use both experimental tasks and partner-report to investigate this further.

In contrast to the qualitative study [14], the positive protective factor of mental resilience was not strongly endorsed by the community sample (24%). Future research could explore if this is a suitable target for future intervention. Also in contrast to the qualitative findings [14], almost half of participants reported talking to their endocrinologist about their mental health with a significant proportion being referred for additional support. Although these are undoubtedly positive findings, this is still a long way from the 88% that experience low mood, and further work is needed to ensure patients are receiving the psychological support they desperately need.

Finally, the current study attempted to account for individual variation in medical history to explore if any factors could predict participants’ scores, finding that GHRT significantly reduced the reported physical impact of AoC. It should be acknowledged, however, that (i) the sample size is relatively small for such an analysis; (ii) the sample was fairly homogenous (only a few individuals were not taking GHRT); and (iii) the overall model was not significant, and therefore interpretation of this significant predictor should be treated with caution. As such, it was difficult to explore these important factors in the current study and this should be an area of priority in future research. A cautious interpretation of the current findings may speak to the fact that GHRT is known to improve physical symptoms [3, 23, 24], while highlighting that the current measure of physical impact also includes the psychological impact of these symptoms, thus this finding adds to the literature demonstrating the importance of GHRT on *psychological*, as well as physical wellbeing [24, 25].

Research has also demonstrated that oxytocin has an important role in psychological functioning [26, 27] and that hypopituitary patients may present with low oxytocin [10–12]. Given the clear overlap in oxytocin’s role and the specific difficulties identified in the current study, further research is needed to explore the potential of oxytocin replacement in AoC [28].

This study adds to the small but growing literature demonstrating the psychosocial impact of AoC. There has been a precedent in the literature to study the psychological impact of childhood-onset craniopharyngioma (CoC), with the assumption that this is greater and more widespread than in AoC. The current results call this into question with comparable effects of CoC [29] and AoC on emotional dysfunction (CoC 40%, current paper 40%) and greater effects of social impairment in AoC (CoC 41%, current paper 56%). Given this, the impetus on future research should be to readdress the balance by focusing on understanding and intervening on the substantial impact observed in AoC.

A strength of this study is utilising a sequential mixed-method design which enhances both the content and ecological validity of the findings. The subscales demonstrated good internal consistency, and the final sample size was comparable to, or larger than, all existing psychological studies [9–12, 14]. Nevertheless, the current study had to employ stringent screening criteria to assess the large initial response, and despite best efforts to utilise automatic coding and validated screening procedures, it is acknowledged that these steps may have introduced selection bias. While following up the detailed lived experiences of those in the UK with data from other UK AoC patients is sensible and, in some ways, preferable, it is also acknowledged that the exclusion of the USA data limits the generalisability of the findings and caps the sample size. An important area for future research will be to systematically assess the generalisability of the questionnaire in a larger, more diverse sample. Relatedly, the current study relied on accurate self-identification of AoC and was therefore unable to account for detailed medical history variables such as histology, extent of hypothalamic involvement, surgical technique etc. Future research should seek to use verified patients such that these clinical details can be used to investigate if any of these factors predict better (worse) outcomes.

In conclusion, this study provides new quantitative evidence that AoC is associated with widespread psychological consequences that meaningfully impact patients’ daily lives. The findings emphasise the need for integrated psychosocial care in AoC management and endocrinologists should be encouraged to discuss patient mental health and facilitate referrals where possible. The results also highlight patient demand for further research which should focus on investigating the issues highlighted here in further detail and longitudinally.

## Supplementary Information

Below is the link to the electronic supplementary material.

Supplementary Material 1

## Data Availability

All the data and code are available online (https://osf.io/3wrpy/?view\_only=9a08260f4f9849b48fc721acc788aaff).
