# Supplementary material for: The psychological impact of adult-onset craniopharyngioma: A follow-up survey
Source: Endocrine. 2026 Feb 16;91(1):81. doi: 10.1007/s12020-025-04497-0 (PMC12909468; doi:10.1007/s12020-025-04497-0)
Supplement: Supplementary file 1 — Supplementary Material 1 [file 12020_2025_4497_MOESM1_ESM.pdf]

Endocrine

The psychological impact of adult-onset craniopharyngioma: A follow-up survey

Katie Daughters[1*]

[1] Department of Psychology, University of Essex, Colchester, UK

*Corresponding author:

Dr Katie Daughters, k.daughters@essex.ac.uk

Supplemental Information 1

USA Data

Overview

The data were analysed in the same manner as the UK data. After reflection on the clinical characteristics, the poor reliability, and observation of the percentage endorsements the decision was made to stop all further analyses.

The Sample

Table S1.1 - Clinical characteristics of the USA sample

Characteristic	
**Age (years)**	48 (33-61)
**Age at Diagnosis (years)**	45 (29-60)
**Sex**	25 (76%) Male
	8 (24%) Female
**Undergone Surgical Resection**	33 (100%)
**Undergone Radiotherapy**	16 (49%)
**Taking Hormone Replacement**	33 (100%)
**Diagnosed Diabetes Insipidus**	16 (49%)
**Taking Growth Hormone**	16 (49%)
**Diagnosed Mental Health Condition**	0 (0%)
**Self-Diagnosed Mental Health Condition**	1 (3%)

Reliability

Although the physical ($\alpha = .63$) subscale achieved good internal consistency, the psychological subscale did not ($\alpha = -.154$). Unfortunately, due to the negative alpha value the data were deemed to be non-reliable, and the data were not analysed further.

Percentage Endorsement

The percentage item endorsement was calculated in the same way as reported in the main paper (see Table S2.1). In general, there was a stronger tendency for responses to aggregate around the central value, and for responses to be more conflicted across items compared to the UK sample.

There was moderate endorsement of a psychological impact of AoC. Almost half reported experiencing low mood (49%, compared to 88% in the UK sample) but generally felt that their diagnosis had not changed the way they felt about themselves (21%, compared to 68% in the UK sample). There were mixed responses regarding anxiety, with only 3% reporting they experienced anxiety, yet 42% saying they do not feel confident in social situations and 45%

stating they had missed social events due to anxiety. The majority said they were good at recognising others' emotions (79%), but were less confident on their own (45%) and 84% also reported crying for no reason. There were also mixed results with their relationships, while only 24% said their relationship with their partner had changed (compared to 68% in the UK sample), 70% said they saw their friends less than they used to (compared to 48% in the UK sample). Finally, only 24% had discussed their psychological wellbeing with their endocrinologist, and only 6% had been referred for further support (compared to 48% and 44%, respectively in the UK sample).

There was moderate endorsement for physical consequences of AoC, however, the findings were also conflicted. About half the sample reported weight gain (54%, compared to 84% in the UK sample), and only 33% had had to make changes to their lifestyle due to weight gain (compared to 64% in the UK sample). While only nine percent reported experiencing fatigue, 57% reported missing events due to fatigue. Headaches appeared to be less of an issue for the USA sample (24%) compared to the UK sample (72%).

Finally, although almost all participants felt that further research into the psychosocial aspects of AoC would be interesting (94%), only 42% thought this research would be useful.

Table S1.2 - Full percentage endorsement for USA participants

	Definitely Agree	Agree	Neither	Disagree	Definitely Disagree
**Theme 1 – Psychological Impact of AoC**					

**I have experienced periods of low mood.**	3	**46**	24	27	0
**I don't feel like myself anymore.**	6	15	36	**42**	0
**I have not felt anxious.***	6	27	**63**	3	0
**I'm confident in social situations.***	3	**36**	18	9	33
**I have missed social events due to anxiety.**	30	15	12	**42**	0
**I have never experienced 'brain fog'.***	9	36	**52**	3	0
**I'm good at recognising other people's emotions.***	**52**	27	18	3	0
**I'm good at recognising my own emotions.***	30	15	**46**	9	0

**I often cry without really knowing why.**	**42**	**42**	9	6	0
**I was more emotional in the period around my surgery, compared to now.**	9	**58**	12	3	18
**My number of friends has not changed.***	6	15	18	**61**	0
**My interactions with my friends are exactly the same.***	3	**70**	21	6	0
**I see my friends less often than I used to.**	6	**64**	18	12	0
**My relationship with my partner has changed.**	12	12	21	**55**	0

**If I feel down, I pick myself up and carry on.***	9	18	**61**	12	0
**I have not felt depressed.***	6	12	24	**58**	0
**I find it easy to cope with having a long-life condition.***	0	9	30	**61**	0
**I have discussed my psychological wellbeing with my endocrinologist.**	3	21	**70**	6	0
**My endocrinologist has asked about my psychological wellbeing.**	9	**73**	3	15	0
**I have not been referred for mental health support.***	9	**76**	9	6	0

Theme 2 – Physical Impact of AoC					
**I have experienced weight gain.**	6	**48**	18	9	18
**I have had to make changes to my normal lifestyle because of my diagnosis/treatment-related weight gain.**	6	27	9	**39**	18
**My weight gain has not affected my self-esteem.***	24	18	15	9	**33**
**I have not experienced fatigue.***	27	**49**	15	9	0
**I have missed social events because I am too tired to attend.**	9	**49**	18	6	18

**I have experienced low libido.**	9	30	18	**42**	0
**I enjoy being intimate with my partner.***	**58**	24	12	6	0
**I experience more headaches than I used to.**	0	24	**70**	6	0
**Theme 3 – AoC Research**					
**I would find more information on the psychological impacts of adult-onset craniopharyngioma interesting.**	0	**94**	6	0	0
**I would find more information on the psychological impacts of adult-onset craniopharyngioma useful.**	0	**42**	36	21	0

*Items that were reverse scored.

Scores may sum to +/- 1 over 100 due to rounding.

Values in bold represent the highest frequency response.

Shaded values represent the general endorsement of the item.

Supplemental Material 2

Table S2.1 – Full percentage endorsement for each item in the survey for the UK sample

	Definitely Agree	Agree	Neither	Disagree	Definitely Disagree
**Theme 1 – Psychological Impact of AoC**					
**I have experienced periods of low mood.**	24	**64**	8	0	4
**I don't feel like myself anymore.**	28	**40**	28	4	0
**I have not felt anxious.***	8	20	24	**32**	16
**I'm confident in social situations.***	12	20	**40**	24	4
**I have missed social events due to anxiety.**	16	**40**	24	12	8
**I have never experienced 'brain fog'.***	4	24	**36**	28	8

**I'm good at recognising other people’s emotions.***	24	**40**	16	20	0
**I'm good at recognising my own emotions.***	24	**44**	12	20	0
**I often cry without really knowing why.**	12	28	24	**32**	4
**I was more emotional in the period around my surgery, compared to now.**	12	**36**	28	20	4
**My number of friends has not changed.***	8	**36**	12	32	12
**My interactions with my friends are exactly the same.***	20	28	12	**32**	8

**I see my friends less often than I used to.**	16	**32**	20	28	4
**My relationship with my partner has changed.**	4	**64**	16	12	4
**If I feel down, I pick myself up and carry on.***	12	12	40	**36**	0
**I have not felt depressed.***	0	**40**	20	28	12
**I find it easy to cope with having a long-life condition.***	8	**36**	24	24	8
**I have discussed my psychological wellbeing with my endocrinologist.**	8	**40**	24	16	12

**My endocrinologist has asked about my psychological wellbeing.**	4	**56**	16	12	12
**I have not been referred for mental health support.***	12	24	20	**32**	12
**Theme 2 – Physical Impact of AoC**					
**I have experienced weight gain.**	20	**64**	8	8	0
**I have had to make changes to my normal lifestyle because of my diagnosis/treatment-related weight gain.**	20	**44**	24	8	4
**My weight gain has not affected my self-esteem.***	8	**40**	16	24	12

**I have not experienced fatigue.***	8	24	16	**40**	12
**I have missed social events because I am too tired to attend.**	8	32	**36**	24	0
**I have experienced low libido.**	12	**36**	32	20	0
**I enjoy being intimate with my partner.***	0	44	44	12	0
**I experience more headaches than I used to.**	20	**52**	20	8	0

Theme 3 – AoC Research

**I would find more information on the psychological impacts of adult-onset craniopharyngioma interesting.**	32	**36**	8	20	4
**I would find more information on the psychological impacts of adult-onset craniopharyngioma useful.**	32	**40**	16	8	4

*Items that were reverse scored.

Values in bold represent the highest frequency response.

Shaded values represent the general endorsement of the item.
